# Successful Surgical Treatment of a Giant Left Ventricular Myxoma Complicated by Infrarenal Abdominal Aortic Occlusion: A Case Report

**DOI:** 10.7759/cureus.87364

**Published:** 2025-07-06

**Authors:** Hiroki Yamazaki, Hiromasa Nakamura, Hiroki Yamaguchi, Tasuku Kadowaki, Takashi Takano

**Affiliations:** 1 Cardiovascular Surgery, Showa Medical University Koto Toyosu Hospital, Tokyo, JPN; 2 Cardiovascular Surgery, Sukagawa Hospital, Sukagawa, JPN

**Keywords:** acute arterial occlusion, cardiac tumor in adults, myxoma complications, myxoma surgical resection, ventricular tumor

## Abstract

A 39-year-old woman presented with acute right leg pain. Computed tomography revealed occlusion of the abdominal aortic bifurcation and right leg arteries. Transthoracic echocardiography showed a mobile left ventricular mass (8 × 30 mm), suspected to be the embolic source. Embolectomy was performed, followed by resection of the giant left ventricular myxoma, confirmed by histology. This rare case of a left ventricular myxoma causing acute aortic and lower extremity occlusion highlights the need for careful consideration of surgical priority and timing in complex embolic events.

## Introduction

Myxoma is the most common heart tumor, with embolism occurring in 30%-40% of cases [1,2]. Obstruction of the abdominal aorta by a giant myxomatous embolus is very rare but associated with high complication and mortality rates [2,3]. We present a case of abdominal aortic occlusion caused by a left ventricular myxoma. Thrombus extraction was performed as an emergency surgery, followed by left ventricular tumor resection six hours after the embolectomy.

## Case presentation

**Figure 1:**
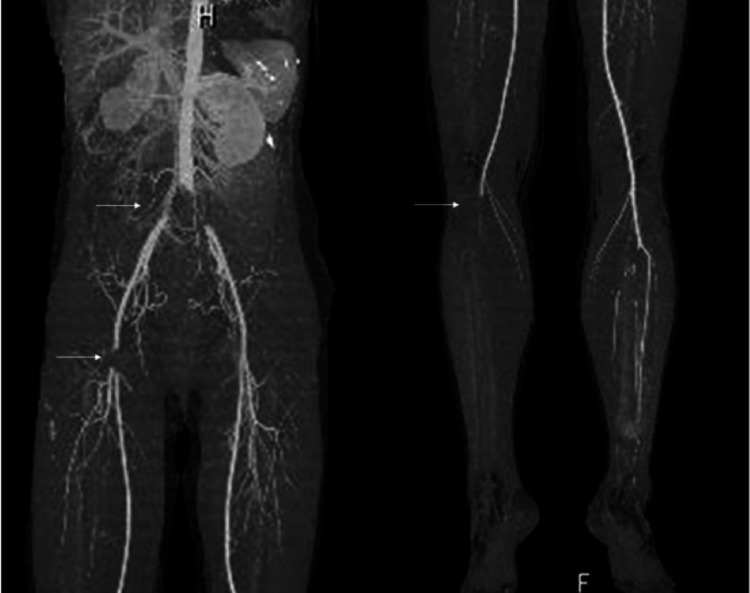
Pre-operative contrast-enhanced CT CT reveals the occlusion of the abdominal aorta, left and right common iliac arteries, right common femoral artery, and right patellar artery, as indicated by arrows. CT, computed tomography

**Figure 2:**
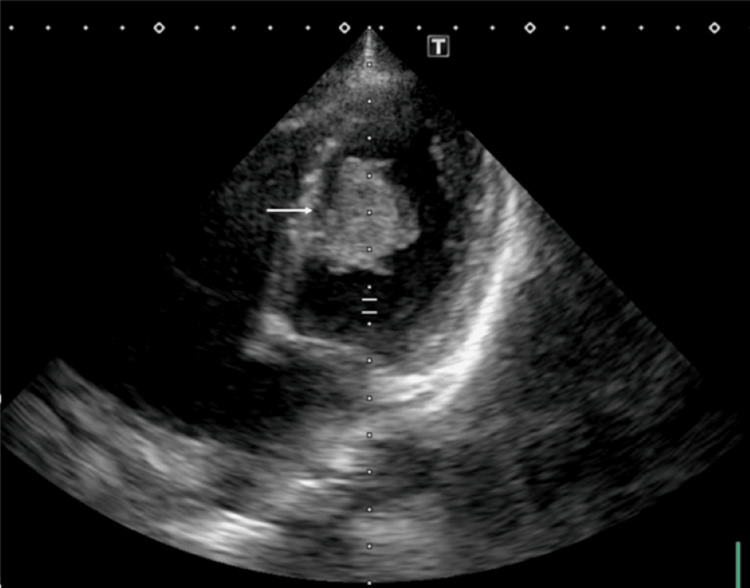
Pre-operative transthoracic echocardiography Approximately 30-mm, pedunculated mass with mobility in the left ventricle (arrow).

A 39-year-old woman with no prior medical history presented with sudden abdominal and right leg pain, leading to the suspicion of acute arterial occlusion at an outside hospital and subsequent transfer to our hospital four hours after the onset of symptoms. Physical examination revealed abdominal pain and a cold, cyanotic right lower extremity with a difficult-to-palpate femoral artery. Enhanced computed tomography (CT) showed obstruction of the abdominal aortic bifurcation, left and right common iliac arteries, right common femoral artery, and right popliteal artery (Figure 1), while transthoracic echocardiography demonstrated a highly mobile giant mass (8 × 30 mm) in the left ventricle (Figure 2).

Laboratory data are shown in Table 1; the electrocardiogram showed sinus rhythm at 78 bpm, and the chest X-ray indicated a cardiothoracic ratio (CTR) of 50% without congestion or effusion.

**Table 1:** Pre-operative laboratory data Abnormal values are indicated in bold. WBC, white blood cell count; Hb, hemoglobin; Plt, platelet count; AST, aspartate aminotransferase; ALT, alanine aminotransferase; BUN, blood urea nitrogen; CK, creatine kinase; CRP, C-reactive protein; FDP, fibrin degradation products; BNP, B-type natriuretic peptide

Test	Result	Normal Range
WBC	13,620 /μL	3,800-10,800 /μL
Hb	11.4 g/dL	11.6-14.8 g/dL
Plt	209,000 /μL	14,000-40,000 /μL
AST	20 U/L	13-30 U/L
ALT	10 U/L	7-30 U/L
BUN	10.5 mg/dL	8-20 mg/dL
Creatinine	0.74 mg/dL	0.46-0.70 mg/dL
CK	178 U/L	41-153 U/L
CRP	0.36 mg/dL	<0.14 mg/dL
D-dimer	0.9 μg/mL	<1.0 μg/mL
FDP	3 μg/mL	<5.0 μg/mL
BNP	57.1 pg/mL	<18.4 pg/mL

A part of the left ventricular mass, suspected as the cause of this acute arterial occlusion, was strongly suspected. The surgical intervention was meticulously planned as follows: firstly, a thrombectomy was performed to salvage the right leg; subsequently, left ventricular tumor resection was also undertaken.

First intervention (after 10 hours of the appearance of symptoms)

Bilateral common femoral arteries were exposed under general anesthesia. After heparinization (6,000 units of heparin), the left common femoral artery was clamped and incised. Then, the central-side embolism was removed using a 4-Fr Fogarty catheter. Thereafter, good flow was obtained. Next, the right common femoral artery was clamped and incised. Similarly, embolectomy was performed using a 4-Fr Fogarty catheter on the central side and a 3-Fr Fogarty catheter on the peripheral side. After achieving good central and peripheral flow, the bilateral common femoral arteries were closed using 6-0 Prolene. The embolus was jelly-like. Fifteen hours after onset (and five hours after embolectomy), compartment syndrome of the right lower limb occurred. Fascial decompression incision was performed by orthopedic surgeons before the heart surgery.

Second intervention (after 18 hours of the appearance of symptoms)

**Figure 3:**
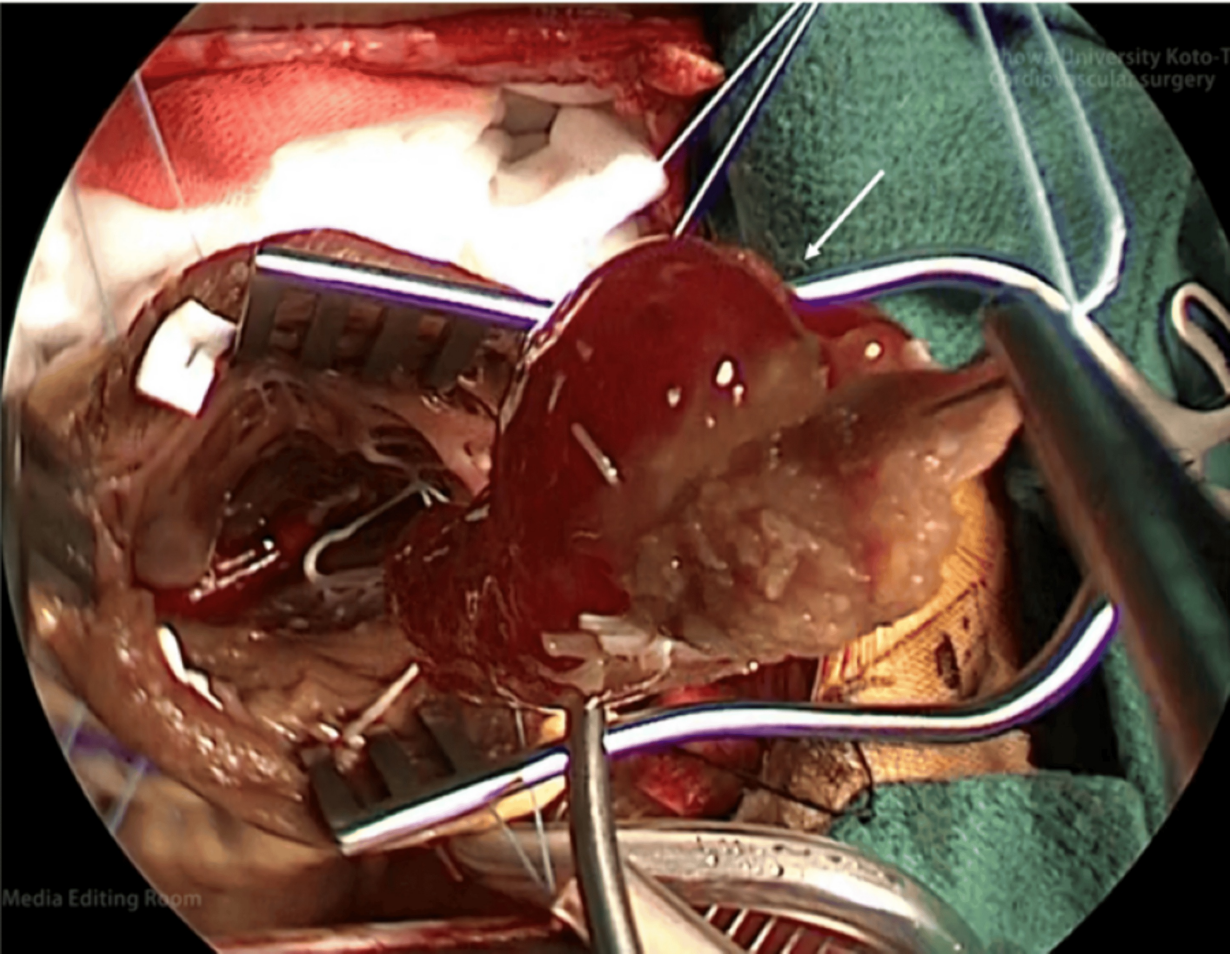
Overall picture of the tumor in the surgical field A jelly-like left ventricular mass of approximately 3 × 3 cm is present in the left ventricle (arrow).

Subsequently, left ventricular tumor resection was performed via median sternotomy. Aortic and bicaval cannulations were used to establish cardiopulmonary bypass. Cardiac arrest occurred due to antegrade cardioplegia (MIOTECTER Cardioplegic Solution®). After making an incision in the ascending aorta and confirming that the tumor had not scattered, an incision was made between the left anterior descending artery and the diagonal coronary artery to reach the left ventricle. A pedunculated, 3 × 3-cm, jelly-like giant tumor with an attachment to the interventricular septum was found (Figure 3). The tumor was resected as a mass from the attachment site. Cryoablation was added to the surgical margin to prevent recurrence after tumor removal. Two Teflon felt strips were sutured horizontally with 1-0 Ethibond mattress sutures to repair the left ventricle wall, followed by double continuous sutures with 2-0 Prolene for reinforcement.

Post-operative course

**Figure 4:**
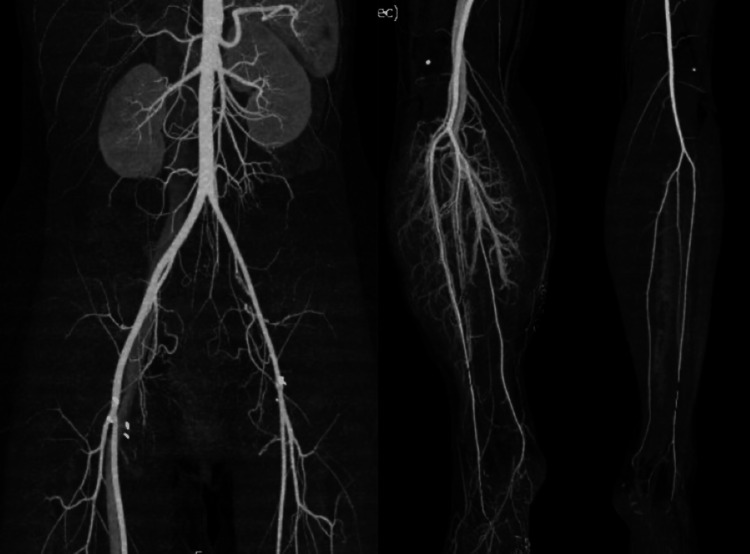
Post-operative contrast-enhanced CT The flow to the limbs was improved. CT, computed tomography

Continuous hemodiafiltration (CHDF) was used because myonephropathic metabolic syndrome (MNMS) occurred (maximum creatine kinase (CK): 70,800 U/L) with compartment syndrome of the right lower extremity postoperatively. Since urine volume was maintained and CK steadily decreased with the use of CHDF, it was discontinued five days postoperatively. Postoperative enhanced CT showed good blood flow results (Figure 4).

**Figure 5:**
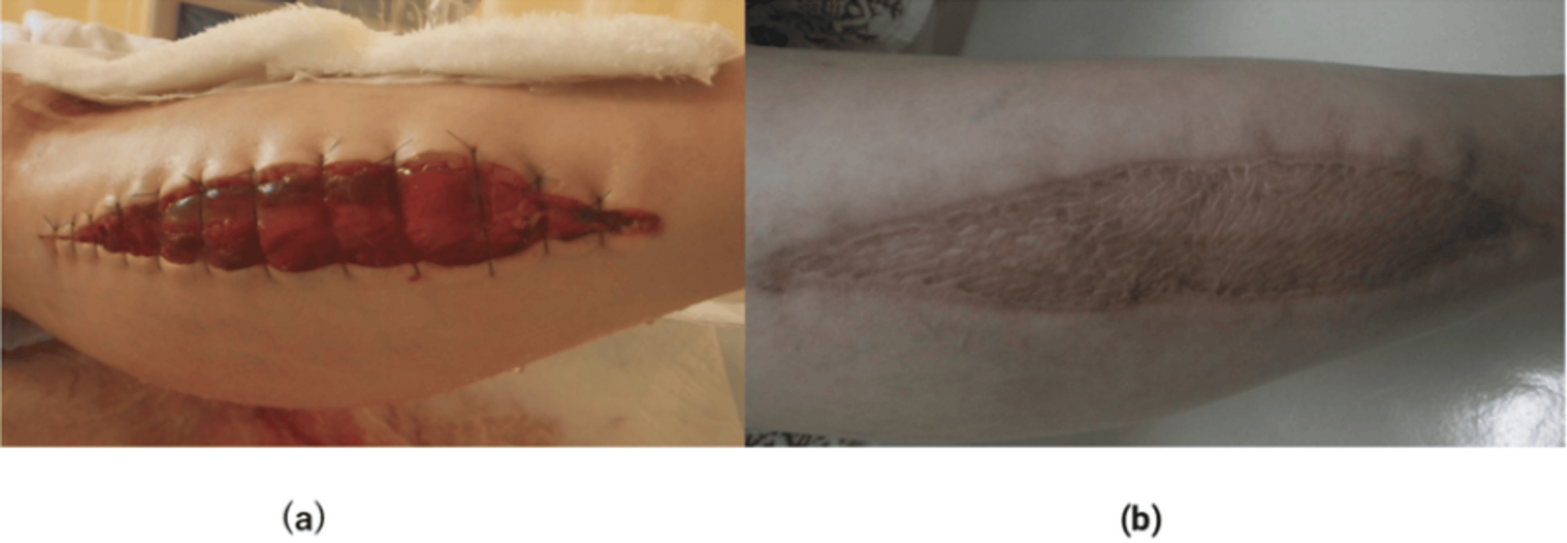
Wound of rhabdomyolysis of the lower limb (a) After fasciotomy and (b) After skin grafting

Vacuum-assisted wound closure therapy was required at the site where fasciotomy was performed; however, the course was stable, and skin grafting was performed on the skin defect by plastic surgeons (Figure 5).

**Figure 6:**
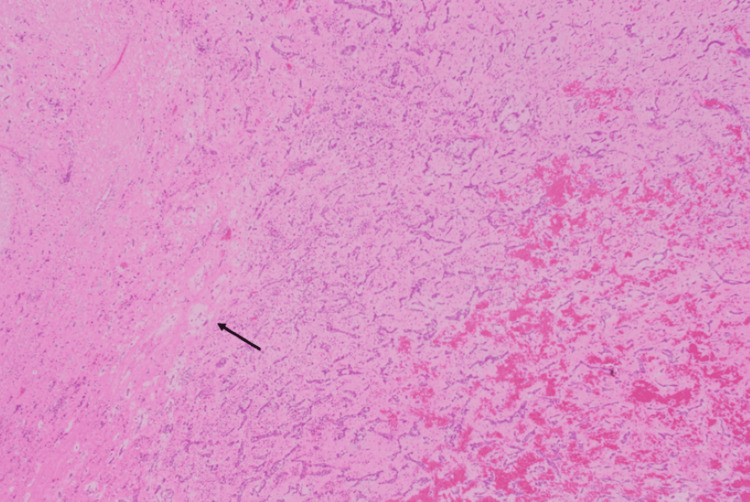
H&E stain Myxomatous changes in heart muscle fibers. The arrow denotes the myxoma cell. H&E, hematoxylin and eosin

No supraventricular or ventricular arrhythmia was observed during the postoperative course. Two years have passed since the surgery, but no recurrence has been observed, and the treatment course is good. Histopathological examination revealed typical findings of cardiac myxoma (Figure 6).

## Discussion

Myxoma is the most common type of heart tumor and occurs in 0.5 out of 1 million people annually [4]. Approximately 80% of myxomas develop in the left atrium; 75% of these originate from the atrial septum, 7%-20% from the right atrium, and the remaining 10% from the right or left ventricle [1]. Due to their mobile nature, myxomas may prolapse through the atrioventricular valves in diastole and return to the atrium in systole. The most common symptoms are those related to mitral valve occlusion, such as congestive heart failure; other symptoms include cerebral infarction, transient ischemic attack (TIA), and peripheral vascular occlusion [1]. An embolism occurs in 30%-40% of patients with myxoma. In particular, those located in the left ventricle pose the greatest risk for morbidity and mortality due to embolization to the systemic circulation (e.g., cervical branch, renal artery, coronary artery) or partial or complete occlusion of coronary circulation [2]. Reports of complete occlusion of the abdominal aorta due to a tumor in the left ventricle are limited [3,5]. 

The potential for ischemia-reperfusion injury and acute compartment syndrome (ACS) is invariably present when limbs exhibiting acute ischemia undergo reperfusion. Ischemia-induced blood flow impairment has been demonstrated to cause changes in endothelial function and hemodynamic function. Furthermore, edema within muscle compartments has been shown to lead to increased interstitial pressure and microcirculatory dysfunction. In the absence of a decrease in compartment pressure, the outcome is inevitable: tissue necrosis. The time until irreversible damage to nerves or muscles occurs varies by location; however, in the lower limbs, it may occur within four hours of complete ischemia onset. A particularly challenging clinical issue is ACS during and after acute thrombolytic therapy, where accurate diagnosis is difficult, and delays in diagnosis have been reported [6]. Therefore, the recent treatment guidelines for acute leg ischemia, published by the European Society of Vascular Surgery, state that rapid fasciotomy is necessary in cases suggesting a high suspicion of ACS or those diagnosed with ACS.

We experienced a case of acute arterial occlusion in the abdominal aorta and lower limbs due to a left ventricular myxoma. The strategy was to perform left ventricular tumor resection after an urgent limb rescue. After five hours of embolectomy, compartment syndrome occurred, and fascial decompression was required. Since the left ventricular tumor was large and highly mobile, a high risk of re-embolism was considered, and left ventricular thrombectomy was performed subsequently.

This case was caused by the sudden onset of abdominal pain and lower limb pain; however, previous reports have described various forms of onset, such as spinal cord ischemia due to blockage of the Adamkiewicz artery, causing lower-limb paralysis [3]. In this case, blood flow in the lower limbs resumed about 10 hours after onset, and compartment syndrome and MNMS developed. If blood flow had been resumed within the golden time (within six hours after the appearance of symptoms), these complications could have been avoided.

Three years after the surgery, no recurrence has been observed. However, recurrence was observed in 7% of patients from three months to 14 years postoperatively, and 87% of them were reported to have a recurrence within five years [7]; therefore, regular follow-up is necessary.

## Conclusions

We experienced a case of acute abdominal aortic and lower extremity arterial occlusion due to a giant left ventricular myxoma. The patient’s life was saved by an emergency embolectomy and left ventricular tumor removal. In similar cases, it is imperative to prioritize the enhancement of blood flow in the lower limbs, with immediate subsequent action aimed at the extraction of the left ventricular myxoma.
